# Helping women veterans quit smoking: a qualitative analysis of successful and unsuccessful attempts

**DOI:** 10.1186/s12905-020-00918-6

**Published:** 2020-03-30

**Authors:** Kristin M. Berg, Stephanie J. Gruber, Douglas E. Jorenby

**Affiliations:** 1 University of Wisconsin Center for Tobacco Research and Intervention, 1930 Monroe St Suite 200, Madison, WI 53711 USA; 2 William S. Middleton Memorial Veterans Hospital, 2500 Overlook Terrace, Madison, WI 53705 USA

**Keywords:** Tobacco cessation, Women veterans, Qualitative research

## Abstract

**Background:**

Tobacco use is the number one cause of death and disability of women in the United States, and our women Veteran population is disproportionately affected. Despite revisions to the Veterans Affairs’ approach to smoking cessation, women continue to smoke at equal or higher rates than men, are prescribed cessation medications less frequently, and are less likely to quit. In this qualitative pilot study, individual interviews with women Veterans revealed their experiences with smoking cessation attempts.

**Methods:**

The lead author conducted semi-structured interviews with 14 women Veterans who were either current or former smokers. Participants gave a narrative account of recent quit smoking attempts. Inductive thematic analysis explored the underlying themes.

**Results:**

Four main themes emerged as important: health and well-being, smoking as an addiction, optimism, and resilience. Health and well-being encompassed physical health, mental health, and financial stability. Women often felt that stability in these key areas made a successful attempt possible. Women with successful quit attempts were more likely to consider tobacco use as an addiction, as well as expressed optimism about their quit attempts. Women with successful quit attempts also demonstrated more resilience to lapses and relapses.

**Conclusions:**

Women Veterans’ quit smoking attempts demonstrate four main themes: baseline health and wellbeing, acknowledging smoking as an addiction, the participant’s optimism towards quitting, and resilience. Patterns were observed within themes with respect to whether the woman was currently quit or had experienced a prolonged quit attempt in the past. Further research is needed to help women Veterans quit smoking.

## Background

Tobacco use is the number one cause of preventable death and disability in the United States [1] and costs the U.S. nearly $300 billion in direct medical expenses and lost productivity [2]. While there has been success in curbing smoking rates in the general population, the United States continues to struggle to help individuals in under-represented population groups quit smoking. Some racial and ethnic minorities, rural populations, individuals with mental health or substance use disorders, and Veterans are among the populations that continue to smoke at higher rates than the general population [3]. The Veteran population is a unique group with particular risks for smoking compared to civilians. The military is a culture in which tobacco use is tolerated, and often encouraged, and efforts to change military and Veteran access to tobacco and smoking culture have been largely unsuccessful [4–7]. Veterans receiving care at the Department of Veterans Affairs (VA) unfortunately struggle with mental health disorders and substance use [8], and cite the challenges of returning to civilian life as additional struggles by which smoking helps them cope [9]. Women Veterans present even more unique challenges, owing partially to high rates of post-traumatic stress disorder [10], mental health disorders [11] and the prevalence of military sexual trauma [12]. Recent surveys confirm that women Veterans, especially those involved in recent conflicts, smoke at significantly higher rates than civilian women (27% compared to 13% in civilians) [11], and even male Veterans (29% women Veterans smoked versus 23% of male Veterans smoked) [13]. With nearly 2 million women Veterans in the United States [14], attention to their epidemic of smoking is critical.

To help combat tobacco use, the VA has implemented a range of smoking cessation resources, including cessation medications, smartphone-based applications, telephone quit lines specifically for Veterans, and Tobacco Cessation Clinics. To better serve women Veterans, the VA has developed women’s health clinics with designated women’s health providers, mental health clinics with dedicated women’s health providers, and gender-specific education materials on tobacco cessation for healthcare providers and patients [15, 16]. Despite these advances, studies have shown that women Veterans are less likely to be satisfied with cessation care received at the VA compared to male Veterans [17] and the organizational measures implemented by the VA were not associated with improved treatment rates or reduced gender differences in smoking [13]. A recent chart review showed that only half of women Veterans who were referred to the William S. Middleton Memorial VA’s Tobacco Cessation Clinic ever followed through with enrollment, but did not examine why [18].

We hypothesize that by better understanding women Veterans’ quit smoking attempts, care processes can be developed to increase the numbers of quit attempts, better serve women Veterans during the quit attempt, and promote long-term success. This project is a small, qualitative, pilot study with women Veterans at a Midwestern Veterans Hospital to begin exploring their previous quit smoking attempts. Inductive thematic analysis was used to explore common themes surrounding successful versus unsuccessful quit attempts.

## Methods

All materials and study procedures were approved by the University of Wisconsin-Madison Health Sciences Institutional Review Board, as well as the William S. Middleton Memorial Veterans Hospital Research and Development Committee. Written informed consent was obtained from all participants prior to participation in this research program. All names presented in this manuscript are pseudonyms to maintain anonymity of the participants.

### Study sample and recruitment

Women Veterans were identified as potential participants based on positive answers to routine tobacco use (conventional cigarette) screening questions asked during clinical visits to VA hospitals or clinics, between January 2013 and June 2015. There were no recruitment efforts based on quit attempt history; potential participants were identified solely on tobacco use screening questionnaires. Letters were mailed to women Veterans identified as using tobacco, describing the research study and inviting their participation in an individual interview to discuss a quit smoking attempt. Interested women were asked to call a VA telephone number and leave their contact information. The lead author called interested participants back and screened for study eligibility, prior to arranging an interview time.

Based on qualitative methodology literature, it was estimated that 20–30 interviews would suffice to reach thematic saturation [19, 20]. Due to limited response rates from initial mailings, multiple mailings and a $50 incentive offer were used to improve recruitment.

### Inclusion/exclusion criteria

To participate in the study, women Veterans had to have made a quit attempt between January 2013 and June 2015, defined as at least 24 h tobacco-free because of actions taken by the participant to avoid tobacco use, with the intent of continuing the quit attempt beyond the 24 h period. Exclusion criteria included a quit attempt that occurred during a hospitalization, being pregnant at the time of the quit attempt, or being enrolled in the Tobacco Cessation Clinic or the integrated smoking cessation clinic within the mental health clinic. Enrollees in these specialized clinics were felt to have had different experiences of care received during their quit attempt than women who did not enroll, and the act of enrolling in the specialized clinics may denote a different background leading to the quit attempt. For the purposes of this preliminary study, it was felt that a general population was more appropriate. Participants who were identified as vulnerable (lacked the capacity to provide consent, did not speak English or were incarcerated), pregnant women, and minors were excluded from the study.

### Interviews

A private, semi-structured interview was scheduled with eligible participants. If possible, the interview was conducted at the VA hospital face-to-face, but a phone interview was offered as an alternative if the participant could not travel to the VA. Prior to the interview, informed consent was obtained from the participant. If the interview was conducted over the phone, the participant was mailed the informed consent paperwork, and the paperwork had to be signed and mailed back prior to scheduling the interview; the paperwork was reviewed with the participant over the phone prior to their interview beginning.

After obtaining informed consent, the interview began with a general request for the participant to “tell [me] about your recent quit smoking attempt.” If the participant had a difficult time openly discussing their experiences or if further clarification was needed, the following sub-questions were asked: “What made you want to quit smoking, did anyone or anything in particular convince you to try to quit?” “How did your healthcare provider work with you, what aspects worked well versus not well?” “What sorts of withdrawal symptoms did you experience, and how did you cope with them?” “What additional strategies did you try during the quit attempt, even without healthcare provider recommendations?” “Were you successful at quitting?” “Why do you think you were/weren’t successful?” and “Tell me about your current use of tobacco.” The questions were designed to be open-ended to allow a narrative account from the patient, thus avoiding unintentional leading questions which might have biased responses. Please see Supplementary file 1 for the full interview guide, which was developed for this study.

The lead author conducted all interviews. Interviews were audio-recorded and transcribed by either the lead author or a research assistant for analysis. Any identifiable information was replaced with de-identified information, and all names were replaced with pseudonyms.

### Analysis

Transcribed interviews were verified by the lead author to be accurate and were read in-depth by authors KB and SG. Using inductive thematic analysis, major themes were identified within the interviews that supported successful or unsuccessful quit attempts. Supporting and refuting evidence was sought in the other interviews. This process continued until no new themes were identified.

With respect to analytic bias, the authors of this paper recognize their positivistic epistemology. As all authors are healthcare professionals trained in advanced smoking cessation techniques, there was an inherent limitation to inductive thematic analysis as it was difficult to avoid making any assumptions about what major themes would unfold. To help support the validity of this analytic technique, authors KB and SG worked independently to identify themes and then discussed their preliminary results to come to consensus agreement. Preliminary results and supportive quotes from the participants were also reviewed with the University of Wisconsin-Madison Institute for Clinical and Translational Research Qualitative Research Group. This is a group of professionals from various healthcare-related backgrounds, led by experienced qualitative researcher Dr. Nora Jacobson. This group reviewed our results and analytic technique, providing an independent validation of the analytic technique.

## Results

**Fig. 1:**
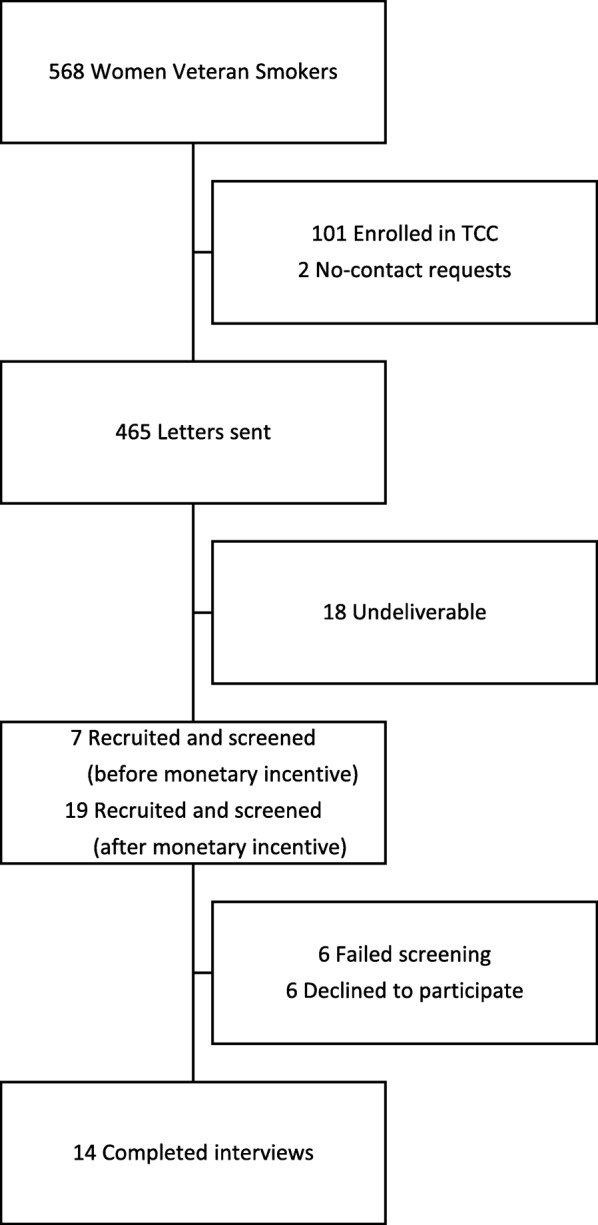
Participant Recruitment

During the January 2013 through June 2015 timeframe, 568 women Veterans screened positive for tobacco use at a clinical encounter. Of those, 101 were excluded for being enrolled in the VA Tobacco Cessation Clinic. Two women were removed from the recruitment mailing list due to a notation that they did not wish to receive solicitations. From the 465 women sent letters, 26 responded as interested, 6 were screened ineligible, and 6 declined to participate. Fourteen women were interviewed, 3 of which were interviewed over the phone. Of the interviewees, 3 women were not smoking at the time of the interview (Fig. 1). An additional two women had previously quit smoking for over one year but had relapsed back to smoking at the time of the interview.

Despite asking women Veterans to discuss one recent quit smoking attempt, women Veterans often discussed multiple quit smoking attempts, comparing and contrasting between attempts as they progressed in their narrative. While not intended, this demonstrated how interconnected quit attempts are and how impactful (either positively or negatively) prior attempts are on future attempts.

Four main themes emerged from women Veterans’ narratives about quitting smoking: health and well-being, smoking as an addiction, optimism, and resilience. (Table 1).

**Table 1:** Themes identified in women Veteran’s quit smoking attempts

	Quotes from Successful Attempts	Quotes from Unsuccessful Attempts
**Health and Well-Being**	“For me not to smoke like that, […] I was in a good relationship, I had a steady income, I had my daughter around me. I had a happier life.” –Roberta, smoking but quit for 9 years previously with nicotine inhaler	“[It] might be a little different if […] I get to a better weight, I get to a better feeling, physical feeling […], and maybe I do something more than walk, maybe I would say ‘OK, well now it’s time.’” –Marie, smoking
**Smoking as an Addiction**	“Everything you do is associated with smoking. […] It’s what makes it hard to quit. […] I needed something. That’s why I went to the doctor to get bupropion.” –Jenny, quit for 3 weeks on nicotine patches	“I don’t see smoking as horrible as alcoholism […] Like ‘Oh, quitting smoking, I can do this. I don’t need to call and whatever.’ That’s just been my mindset.” –Jessica, smoking
**Optimism**	“I have more resources like to be able to buy things […] that are good for me, and now […] I can afford to try and cope. The varenicline helps take that edge off […] because I feel great. I wish I could stay this way all the time.’” –Christy, quit for 2 months on varenicline	“If I’ve tried to quit in the past, […] this sounds really pessimistic, but something always seems to go wrong. […] Which, you know, aggravates me to the extent where I’m just going to smoke again and everything will be OK’” –Jessica, smoking
**Resilience**	“[Slipping] doesn’t make you a terrible person. If you, you know, slip, […] you can still get back on track.’” –Samantha, quit for 2 years without medications	“You just feel like, well, kind of just, throw your hands up. You’re like ‘well, I already fell, […] I’m already doing it now so I can’t say three months anymore.’” –Rachel, smoking

**Each theme is accompanied by a representative quote from successful attempts as well as unsuccessful attempts**

### Health and well-being

This theme comprised a triad of physical health, mental health and financial stability. Thirteen of the fourteen women discussed their health and stability as it related to quitting smoking. Of the five women who were either quit at the time of the interview or who had quit previously for a sustained period, four commonly noted that the quit attempt was only possible because their life was under a perceived sense of control. They frequently noted that they felt well physically, had reduced stressors in their life, and felt financially stable; enabling them to quit smoking. One additional woman who was smoking at the time of the interview but previously quit for 2 weeks, reported that when she did quit, she was working, and things were “going well.” Of the eleven women who were smoking at the time of the interview, four women quit in the setting of a significant life event like a parent’s death, upcoming surgery, pregnancy, or financial strain. Four women predicted that if they felt better (for example, having more time to exercise), had less stress (for example, after graduation), or better finances, that they would be able to quit smoking. There were four women who had opposing points of view: two stated that because they felt relatively healthy, they didn’t feel the need to quit smoking. One woman noted that because she had so many medical concerns, she didn’t feel the need to quit because she knew she was “not going to have a good old age,” and one woman noted that a new medical diagnosis caused so much stress it made her smoke more.

### Smoking as an addiction

All fourteen women spoke about smoking as either an addiction or a habit. Of the five women who were currently quit or who had quit for greater than 1 year previously, four spoke very openly about smoking as an addiction. They purposefully sought treatment options, either in the form of approved smoking cessation medications or using principles they learned in alcohol treatment groups to help them quit smoking. They were more likely to equate tobacco use to alcohol use/abuse, one woman stated “I can’t restrict myself [number of cigarettes smoked daily], it’s like being an alcoholic, eventually you’re back to smoking more.” Of the eleven women who were currently smoking, five felt that their smoking was more habit than addiction. One woman had a difficult time identifying triggers to smoke, but five women spoke openly about their triggers. These women often planned to avoid triggers during a quit attempt, or purposefully keep themselves busy so as not to notice the lack of smoking. Some did use medications but referenced stopping these if they felt they weren’t working. Three women expounded more on the loss of smoking. They noted that the hardest part was filling the void of losing a cigarette, feeling that they “lost a friend.” They felt that taking away smoking was a more difficult lifestyle change because it was a subtraction instead of an addition.

### Optimism

Ten women shared statements that were reflective of their optimism about quitting smoking, and this theme tied heavily into the planning that went into a quit attempt, and their views on smoking as an addiction. For the three women not smoking at the time of the interview, one reflected that she knew her quit attempt would work because she had turned her life over to God. She was also using many of the principles she had learned in alcohol treatment, and fully embraced the idea that smoking is an addiction. The other two women shared multiple trials of medications to help them quit smoking, advanced planning to avoid smoking during upcoming stressful life events, and even logging smoking in a diary to understand her triggers and ways to overcome them. The women who were smoking at the time of the interview expressed a lot of frustration over past quit attempts. One expressed that she had goals but doesn’t know how to reach them, one was frustrated at the nicotine patches and returned to smoking because of the difficulties with patches (including not sticking and their conspicuous nature). One woman noted that her heart “just wasn’t in it,” and four women mentioned fears about quitting including being afraid to tell others about a quit attempt, fears about the repercussions of quitting like weight gain or insomnia, and fears of failing.

### Resilience

This last theme focused on how women reacted to a lapse (temporarily returning to smoking) or a full relapse back to smoking. For the three women who were quit at the time of the interview, two expressed guilt and frustration over lapses; however they referenced their acknowledgement of smoking as an addiction, as well as their treatment plans, which enabled them to recycle the quit attempt. For example, one woman noted that she ran out of nicotine patches for a week on vacation and returned to smoking but was able to purchase more patches upon returning home and quit again. The woman who reported turning her life over to God likened lapsing to a child misbehaving, and that she would re-commit herself and was able to go back to quitting. For the women who were smoking at the time of the interview, their resilience to lapsing and relapsing was more limited. Four women expressed guilt, frustration, or disappointment upon returning to smoking. One stated she feels like “throwing her hands up” when she lapses, thus returning to smoking instead of trying to quit again. Two women expressed the opposite reaction to lapsing. They felt relief at being able to cope with the stressful life events or mental health struggles that prompted their return to smoking.

## Discussion

The purpose of this qualitative study was to begin to understand factors that are associated with successful versus unsuccessful quit smoking attempts in women Veterans at a Midwestern VA. We found four main themes that resonated throughout the interviews with regards to smoking cessation: health and well-being, smoking as an addiction, optimism, and resilience. From the three women who were quit at the time of the interview, plus the additional two women who had quit for substantial periods previously, there were patterns that were observed surrounding the themes with respect to the success of their quit attempts.

Seeming to set the stage for a quit attempt was the theme of health and well-being. It is well known that women Veterans struggle with health and well-being: surveys of physical health factors demonstrate that Veterans have significantly lower health scores than civilians [21]. In addition, women Veterans have lower levels of social support than their male counterparts [21], and higher rates of mental health conditions compared to male Veterans (particularly non-post traumatic stress conditions) [22]. Poorer physical and mental health combined with lower levels of social support, lead to a distinct disadvantage that women Veterans have with regards to this baseline theme.

The themes of addiction and optimism tied closely to one another. The divide between addiction and habit is unsurprising: historically, public health officials considered smoking as a habit that only needed strong will-power to overcome [23]. While healthcare officials have worked to overcome this historical viewpoint and promote purposeful treatment, two women in this study specifically noted that they could overcome their smoking with will power. One woman even referenced her primary care provider echoing that sentiment. A key tenant of the 2008 Treating Tobacco Use and Dependence Clinical Practice Guideline is recognizing that tobacco use is a chronic disease, necessitating dedicated treatment [1]. Women who took purposeful steps to treat their tobacco use had a more positive outlook when discussing their quit attempts. An analysis of the Women’s Health Initiative project investigated the link between optimism and smoking cessation. While optimism did not predict smoking cessation after controlling for socioeconomic factors, the authors felt that socioeconomic factors and optimism were strongly linked. When socioeconomic factors and demographic covariates (also frequently linked to socioeconomic factors) were taken out of the model, higher optimism scores did predict higher rates of smoking cessation [24].

The final theme, resilience, tied in closely with the other themes and influenced women Veterans’ perceptions about quitting smoking in the future. The sentiments of guilt, frustration, and disappointment in the setting of lapses echoed by women in this study are consistent with the abstinence violation effect theory characterized by Marlatt [25], and it has been demonstrated that diminished self-efficacy is a driver of relapse in smokers trying to quit [26]. Compounding this is the military culture of teamwork, and not letting team members down, potentially magnifying these findings.

These themes and the patterns that were observed with respect to successful and unsuccessful quit attempts, may help illuminate areas to further investigate in efforts to help women Veterans quit smoking. There are limitations to this pilot study. Primarily, this was a small sample from a defined population, in one geographic area. Consequently, no causation or correlation should be ascertained from this study with respect to the patterns observed. While no information was gathered on participant backgrounds or their tobacco use patterns, this qualitative project intended to elicit an understanding of the particular lived experiences of these women Veterans. The authors particularly wanted to maintain anonymity of the participants, but also wanted to refrain from making over-reaching conclusions regarding patterns emerging from participant background information or tobacco use patterns. Further studies are needed to confirm whether these particular themes are generalizable to larger, more heterogeneous populations. Due to the limited initial response rates, a small stipend was offered to those women who completed an interview. This compensation may have biased the types of participants who responded; socioeconomically disadvantaged individuals are more likely to smoke and have a harder time quitting [2]. Some interviews were conducted over the phone, if the participant was unable to travel to conduct the interview in person. While concern may arise about different interview techniques dependent upon interview modality, the authors felt that availing a telephone interview avoided a more substantial bias against participants limited in their means to travel. The VA Hospital where interviews took place serves patients from 20 counties, spanning two states, so some patients face a significant commute in to the VA Hospital. There were no substantial trends or changes in themes from women interviewed over the phone versus in person. Finally, there was a trend noticed that some women called the study telephone line and clarified that they had quit smoking with respect to the letters received. Despite best efforts to enroll both smokers and former smokers, recruitment letters may have been interpreted as recruiting only smokers. Despite these limitations, if these results are replicated in a larger, more heterogeneous population, these themes could inform future clinical changes to help women Veterans quit smoking.

## Conclusions

Women Veterans’ quit smoking attempts demonstrate four main themes: baseline health and wellbeing, acknowledging that smoking is an addiction, the participant’s optimism towards the attempt, and their resilience. Patterns emerged within these themes with respect to whether the woman was currently quit or had experienced a prolonged quit attempt in the past. These themes need further investigation and confirmation, but ultimately may provide better understanding of ways providers can help women Veterans quit smoking.

## Supplementary information

**Additional file 1.** Interview Guide.

## Data Availability

The datasets generated and/or analyzed during the current study are not publicly available due to privacy concerns given the limited sample size but are available from the corresponding author on reasonable request.

## Abbreviations

VA: Department of Veterans Affairs
