# Supplementary material for: Helping women veterans quit smoking: a qualitative analysis of successful and unsuccessful attempts
Source: BMC Womens Health. 2020 Mar 30;20:63. doi: 10.1186/s12905-020-00918-6 (PMC7106739; doi:10.1186/s12905-020-00918-6)
Supplement: Supplementary file 1 — Additional file 1. Interview Guide. [file 12905_2020_918_MOESM1_ESM.docx]

Supplementary File – Interview Guide

| Opening question | Please tell me about your recent quit smoking attempt. |
| --- | --- |
| Probing reasons for quitting | What made you want to quit smoking, did anyone or anything in particular convince you to try to quit? |
| Probing healthcare experiences | How did your healthcare provider work with you, what aspects worked well versus not well? |
| Probing withdrawal experiences | What sorts of withdrawal symptoms did you experience, and how did you cope with them? |
| Probing other quitting strategies used | What additional strategies did you try during the quit attempt, even without healthcare provider recommendations? |
| Probing outcomes of quit attempts | Were you successful at quitting? Why do you think you were/weren’t successful? |
| Probing current smoking status | Tell me about your current use of tobacco. |
